# The antioxidant and therapeutic effects of *Malva sylvestris* extract on testicular tissue and sperm quality in varicocele-induced adult Wistar rats

**DOI:** 10.1186/s12610-025-00271-4

**Published:** 2025-06-16

**Authors:** Fatemeh Koohkan, Mahnaz Azarnia, Latifeh Karimzadeh Bardeei

**Affiliations:** Department of Animal Biology, Faculty of Biological Sciences, Kharazmi University, Tehran, Iran

**Keywords:** Infertility, *Malva sylvestris*, Oxidative stress, Sperm, Testis, Varicocele, Infertilité, *Malva sylvestris*, Stress oxydatif, Spermatozoïdes, Testicules, Varicocèle

## Abstract

**Background:**

Varicocele is a major male infertility issue, and *Malva sylvestris* shows promise as a treatment due to its antioxidant properties. The present study evaluated the protective effects of *Malva sylvestris* on testicular health, sperm quality, and oxidative stress-related gene expression. Adult male Wistar rats were randomly assigned into five groups (*n* = 8): the control group, the varicocele model group, the varicocele group with partial occlusion of the left renal vein treated with 750 or 1500 mg/kg *Malva sylvestris* for 21 days, and a surgical sham group. The epididymal content and Histological analyses of animals testicular tissue were examined to evaluate fertility parameters, and qRT-PCR was employed to determine the expression of *SIRT1*, *FOXO1*, *NRF2*, *NF-κB*, and *TGF-β* genes.

**Results:**

Varicocele leads to the induction of apoptosis, the occurrence of DNA damage, a reduction in *SIRT1* and *FOXO1*, and an increase in *NRF2*, *TGF-β*, and *NF-κB* gene expression. According to Histological morphometric analysis, treatment with Malva sylvestris showed the increased thickness of Mean Seminiferous Tubule Diameter and Epithelial Thickness in the spermatogenic epithelium, as well as the presence of a greater number of germ cells and mature sperm. In this study, *Malva sylvestris* treatment showed antioxidative effects since it upregulated the expression of *SIRT1* and *FOXO1* genes and downregulated the expression of *NF-κB*, *NRF2*, and *TGF-β* genes.

**Conclusion:**

Based on the findings, *Malva sylvestris* as cytoprotective agent modulate key antioxidant pathways, including upregulation of SIRT1 and FOXO1 (linked to cellular repair and OS resistance) and downregulation of pro-inflammatory mediators; then, *Malva sylvestris* as a promising natural antioxidant for managing varicocele-related infertility, offering a potential adjunct or alternative to conventional therapies.

## Résumé

**Contexte:**

La varicocèle est un problème majeur d’infertilité masculine, et *Malva sylvestris* est prometteuse en tant que traitement, en raison de ses propriétés antioxydantes. La présente étude a évalué les effets protecteurs de *Malva sylvestris* sur la santé testiculaire, la qualité du sperme et l’expression des gènes liés au stress oxydatif. Des rats Wistar mâles adultes ont été répartis au hasard en cinq groupes (n = 8) : groupe témoin, groupe modèle varicocèle, groupe varicocèle avec occlusion partielle de la veine rénale gauche traité avec 750 ou 1500 mg/kg de *Malva sylvestris* pendant 21 jours, et groupe simulé chirurgicalement. Le contenu épididymaire et les analyses histologiques de tissu testiculaire des animaux ont été examinés pour évaluer les paramètres de fertilité, et la qRT-PCR a été utilisée pour déterminer l’expression des gènes *SIRT1, FOXO1, NRF2, NF-κB *et* TGF-β.*

**Résultats:**

La varicocèle entraîne l’induction d’une apoptose, l’apparition de dommages à l’ADN, une réduction de *SIRT1* et *FOXO1*, et une augmentation de l’expression des gènes *NRF2*, *TGF-β* et *NF-κB*. Selon l’analyse morphométrique histologique, le traitement par *Malva sylvestris *a montré une augmentation de la moyenne de l’épaisseur du tube séminifère et de l’épaisseur épithéliale de l’épithélium séminifère, ainsi que la présence d’un plus grand nombre de cellules germinales et de spermatozoïdes matures. Le traitement par *Malva sylvestris* a montré des effets antioxydants puisqu’il régulait à la hausse l’expression des gènes* SIRT1* et *FOXO1* et à la baisse l’expression des gènes *NF-κB*, *NRF2* et *TGF-β*.

**Conclusions:**

Sur la base des résultats, *Malva sylvestris*, en tant qu’agent cytoprotecteur, module les principales voies antioxydantes, y compris la régulation à la hausse de *SIRT1* et *FOXO1,* et la régulation à la baisse des médiateurs pro-inflammatoires. *Malva sylvestris,* en tant qu’antioxydant naturel prometteur pour la gestion de l’infertilité liée à la varicocèle, offre un complément potentiel ou une alternative aux thérapies conventionnelles.

**Graphical Abstract:**

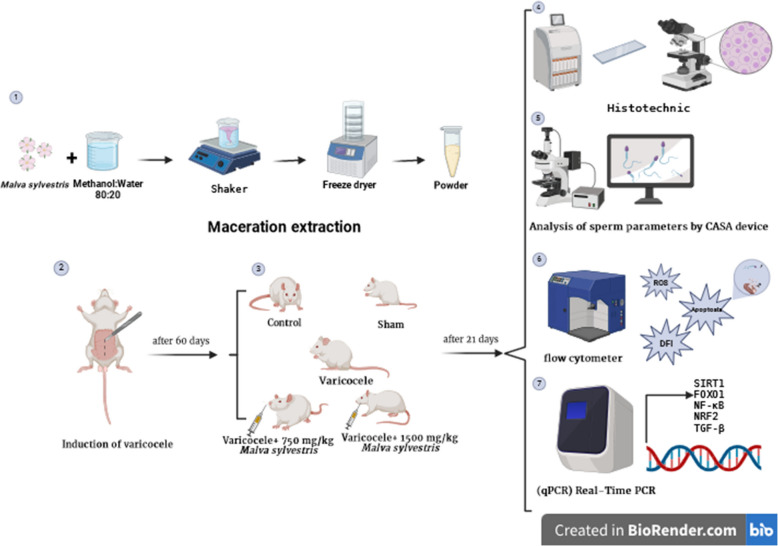

## Introduction

The primary function of the male reproductive system is the generation of spermatozoa and hormones. Male infertility can be caused by various factors, particularly deviations in sperm parameters, which represent as spermatogenic dysfunctions, such as the complete absence of sperm (azoospermia), diminished sperm count (oligozoospermia), anomalous sperm morphology (teratozoospermia), or impaired sperm motility (asthenozoospermia). It is worth noting that the prevalence of male factor infertility varies geographically, ranging from 20 to 70% [1].

Varicocele, characterized by the dilation of veins that carry blood from the testicles, spermatic veins and the pampiniform plexus, is a common cause of infertility in men seeking treatment at infertility centers. Varicocele is prevalent in 11.7% of couples and affects 15% of the general male population. It is the cause of infertility in 25.4% of infertile men, occurring in 35% of men with primary infertility and 75% of men with secondary infertility [2–4].

Approximately 30 to 80% of sterile males exhibit levels of reactive oxygen species (ROS) in their semen that exceed the levels of antioxidants present, which in turn impacts sperm motility and functionality and contributes to the development of varicocele disease. Hypoxia and thermal stress are the primary elements responsible for the increased generation of ROS within germ cells, consequently interfering with the maturation of spermatocytes and the normal operation of epididymal cells. This imbalance leads to DNA damage, which triggers the initiation of the apoptotic pathway, resulting in a decreased sperm count and ultimately leading to male infertility [5–7].

The previous studies underscored that oxidative stress is a central mediator of sperm dysfunction via DNA damage, lipid peroxidation, mitochondrial impairment, and apoptosis; ROS (e.g., superoxide anion, hydrogen peroxide) directly attack sperm DNA, leading to strand breaks and base modifications. High DNA fragmentation reduces fertilization potential and increases the risk of embryonic abnormalities. on the other hand, ROS target polyunsaturated fatty acids (PUFAs) in sperm membranes, triggering lipid peroxidation. Loss of membrane fluidity and acrosome integrity, impairing sperm motility and egg-binding capacity. Also Sperm rely on mitochondrial ATP for motility. Excessive ROS disrupts mitochondrial electron transport, reducing energy production and leading to Asthenozoospermia (reduced sperm motility) and premature sperm exhaustion. ROS activate pro-apoptotic pathways (e.g., Bax/Bcl-2 imbalance, caspase-3) in germ cells, leading to premature cell death and Oligozoospermia and disrupted spermatogenesis. ROS impair Leydig cell steroidogenesis, lowering testosterone production. Testosterone deficiency further exacerbates spermatogenic failure [8–10].

Microsurgery is currently considered the most effective approach for performing varicocelectomy, as it is associated with fewer complications and better pregnancy results [11]. Nevertheless, as surgical interventions are financially challenging for some individuals [12], various types of medications, such as selective estrogen receptor modulators and antioxidants, are used as an alternative to treat varicocele. The use of antioxidants derived from medicinal plants is one approach for reducing oxidative stress levels and enhancing fertility [13, 14].

*Malva sylvestris* (M.S) is one of these medicinal plants which has long been used in traditional herbal medicine and cosmetic procedures [15]. The physiological function of the foliage of M.S is contingent upon various chemical components, such as polyphenols, vitamins C and E, beta-carotene, gossiptin 3, and glucoside, which play a central role in its biological efficacy [16]. The molecular mechanism by which antioxidants derived from M.S alleviate varicocele involves modulation of SIRT1, FOXO1, and pro-inflammatory mediators; addressing oxidative stress and inflammation—key contributors to varicocele pathogenesis. Antioxidants (e.g., flavonoids, phenolic acids) in M.S enhance SIRT1, a NAD⁺-dependent deacetylase. This may occur via increased NAD⁺ availability or direct compound-SIRT1 interaction. SIRT1 deacetylates and activates FOXO1, amplifying its antioxidant functions in testicular tissues. SIRT1 deacetylates NF-κB subunits (e.g., p65), inhibiting their nuclear translocation and transcriptional activity, thereby suppressing pro-inflammatory genes. By inhibiting NF-κB via SIRT1, M.S antioxidants reduce expression of cytokines (TNF-α, IL-6), COX-2, and iNOS, curtailing inflammation. SIRT1-FOXO1 axis enhances cellular resilience to oxidative stress, while NF-κB inhibition reduces inflammation. This dual action protects spermatogenesis and testicular function in varicocele [17–20].

The present study aimed to investigate the protective effects of M.S on alleviating testicular injury in an experimental varicocele model and clarify the precise underlying mechanism of action; The objective of this study is to advances prior work by demonstrating not only histological improvements (e.g., reduced seminiferous tubule degeneration) but also functional recovery in sperm quality (motility, count, morphology), bridging the gap between antioxidant intervention and reproductive outcomes.

## Materials and methods

### Preparation and evaluation of the antioxidant content of M.S extract

M.S flowers were collected from Karaj, Alborz Province, Iran. First, the leaves were separated, desiccated at room temperature, and finely ground. The resulting powder was then placed in 2 L 96% ethanol (Razi, Iran) and stored at 25 °C for 48 h. Subsequently, the mixture was agitated by a shaker (Hoilph, Germany), filtered, and centrifuged at 1000 × g for 8 min (Hanil, South Korea). After the dissolved solvent was evaporated at room temperature, the extracted powder was refrigerated at 4 °C until further analysis. The DPPH test method was used to assess the antioxidant potential of *M.S*. To formulate a DPPH solution, 4 g of pure powder was dissolved in 100 mL of methanol. Then, 6 mL of DPPH was added, and the mixture was allowed to rest for 30 min at 37 °C. Finally, the absorbance of each sample was measured at 517 nm, and the percentage of inhibition of free radicals was calculated using the following formula: I% = (A blank-A sample/A blank) × 100. The reference sample consisted of 3 mL of *M.S* powder and 3 mL of methanol.

### Experimental design and animals

Forty male Wistar rats weighing 200 to 250 g were sourced from the Animal House of Kharazmi University and kept in a controlled environment (22 ±  2 °C, 45–50% humidity, and a 12-h light/dark cycle) with access to water and standard chow ad libitum. All animal experiments comply with the ARRIVE guidelines and carried out in accordance with the National Institutes of Health guide for the care and use of Laboratory animals. The study was approved by the Ethics Committee of Kharazmi University under code IR.KHU.REC.1402.091. After a one-week acclimatization under standard conditions, the rats were divided into five groups (*n* = 8). Group 1 (control) had no varicocele, group 2 had induced varicocele, and groups 3 and 4 (varicocele + M.S) received *M.S* at 750 and 1500 mg/kg (intraperitoneal), respectively, for 21 days following varicocele induction for eight weeks [21–23]. A surgical sham group (group 5) also underwent all procedures except spermatic vein ligation. However, data from this group were excluded from comparative analysis due to a lack of significant difference from the control group.

### Procedures for varicocele induction

Male rats were induced with experimental varicocele using Turner’s method [24] under sterile conditions at room temperature (25°C), as described previously. The rats were anesthetized intraperitoneally with 50 mg/kg ketamine and 10 mg/kg xylazine [25]. A 2–4 cm vertical incision was made along the midline of the abdomen to access the left internal spermatic vein and the left renal vein of the inferior vena cava. The surgical procedure continued with creating a tunnel near the left renal vein and separating the vein from the surrounding fatty tissues. This was followed by partial occlusion of the renal vein through suturing with a 4–0 nonabsorbable silk suture with the assistance of a rod. Upon completion of the suturing, the procedure was ended by withdrawing the auxiliary rod and closing the incision layers by stitching. After an experimental period of eight weeks, all the animals were euthanized through CO_2_ gas inhalation, and the epididymal tissue and testicles were extracted.

### Sperm parameter analysis

The epididymal tubes were dissected in 1 mL of culture medium. Afterward, 10 μL of the solution was placed on a Neubauer slide, fixed with 2% formalin, and examined by the enumeration of five fields at 400 × magnification using a microscope (Zeiss, Germany).

For viability assessment, an aliquot of 5 μL of sperm suspension was placed on the slide, and 2.5 μL of 1% eosin was added to it. Following a 30-s incubation at 37 °C, 2.5 μL of 10% nigrosin was added to the suspension. Subsequently, 1000 viable (unstained) and nonviable spermatozoa (red) were counted under a microscope, and 5 μL of the suspension was placed on a Leja slide with a depth of 20 μL. The slide was preheated to 37 °C prior to assessing the motility rate and other sperm characteristics using Computer-Assisted Sperm Analysis (CASA, Eclipse E-200 microscope, Nikon Co., Tokyo, Japan). Sperm motility parameters consisted of Curvilinear Velocity (VCL), Average Path Velocity (VAP), Straight-Line Velocity (VSL), Linearity Index (LIN), Straightness Index (STR), Wobbling Index (WOB), Beat Cross Frequency (BCF), and Amplitude of Lateral Head displacement (ALH) [26]. Furthermore, progression assessment was conducted according to WHO4 and WHO5 criteria, considering factors such as velocity, head area, and round cells.

### Histopathological evaluations

Varicocele was induced through ligation of the left renal vein in rats, and the outcomes were assessed after eight weeks. After all the animals were anesthetized using previously outlined procedures, their testes were excised for further examination. The left testis was carefully dissected, rinsed with cold saline, and then immersed in a 10% formalin solution for 24 h. The tissue samples were subsequently dehydrated through a series of increasing alcohol concentrations, followed by clarification with toluene. Thin transverse Sects. (4 μm thick) were mounted on glass slides and stained with hematoxylin and eosin (H&E). The histopathological alterations within the testicular tissue were then assessed under a light microscope. For quantitative assessment of testicular morphometry, 100 seminiferous tubules exhibiting circular or maximally round cross-sectional profiles were selected. Tubule diameter was measured as the minor axis using ImageJ software. MSTD was calculated as:

$$\text{MSTD}=\sum (\text{diameters of all measured tubules})/\text{Number of tubules measured}$$

Epithelial thickness was defined as the perpendicular distance between the basement membrane and luminal edge (excluding the lumen). Measurements were taken at five equidistant points per tubule using ImageJ’s straight-line tool, averaged per tubule, and MSET derived as [27]:

$$\text{MSET}=\sum (\text{average epithelial thickness per tubule})/\text{Number of tubules measured}$$

### Sperm chromatin structure assay

First, semen samples were thinned at a ratio of 1:10 with phosphate-buffered saline (PBS; pH 7.4) to examine the quality of sperm chromatin. Next, they were centrifuged at 2300 rpm for 5 min at 4 °C to eliminate seminal plasma. Afterward, they were treated with 70% ethanol for 10 min and went through centrifugation and suspension in PBS again. A total of 10,000 sperm cells were preserved at 28 °C to further analyze their chromatin structure using a mixture of 2 mg/mL acridine orange, 0.15 M NaCl, 126 mM Na2HPO4, 1 mM ethylenediaminetetraacetic acid, and 37 mM citric acid buffer at a pH of 7.4. Finally, the sperm cells were stained, and Sperm Chromatin Structure Assay (SCSA) was conducted to examine the DNA fragmentation index (DFI%) and the percentage of sperm with high-density staining (HDS%) using a flow cytometer [28–30]. In SCSA, a DFI of ≥ 25% was considered abnormal [31].

### Investigation of the ROS content in epididymal tissue

To measure ROS levels, flow cytometric assessment was utilized with 2',7'-dichlorofluorescein diacetate (DCFH-DA) to detect hydrogen peroxide (H_2_O_2_) and dihydroethidium (DHE) to identify superoxide anion (O_2_). The suspensions derived from epididymis in the previous step were incubated with DCFH-DA (10 mmol) and DHE (1.25 μmol) for 15 min at 37°C. The samples were then centrifuged at 350 rpm in the dark until the incorporation of probes into the cellular membranes. After a triple wash with PBS, DCFH was transformed into dichlorofluorescein (DCF), and DHE was transformed into 2-hydroxyethidium (HE). The samples were then analyzed using a flow cytometer (BD Immunocytometry Systems, San Jose, CA, USA), which measured green fluorescence of DCF within the range of 500–530 nm in the FL-1 channel and red fluorescence of HE within the range of 590–700 nm in the FL-2 channel with an excitation wavelength of 488 nm and an emission wavelength of 530 nm [32].

### Examining the mitochondrial membrane potential of testicular tissue with JC-1 dye

To measure the mitochondrial membrane potential (MMP), which indicates the activity of this intracellular organelle, flow cytometry was used. The MMP was assessed using a lipophilic dye (5,5′,6,6′-tetrachloro-1,1′,3,3′tetraethyl-benzimidazolylcarbocyanine iodide) that selectively entered mitochondria. A sample containing 1 × 10^6^/mL sperm was incubated with JC-1 in the dark for 10 min at 37 °C under 5% CO_2_ pressure within an incubator, followed by a PBS wash. Changes in membrane potential led to the transition of JC-1 dye from green to orange, allowing differentiation between normal and damaged cells based on the presence of orange granules [33, 34].

### Analysis of sperm apoptosis by flow cytometry

Membrane translocation of phosphatidylserine (PS) and membrane integrity were assessed using the Annexin V technique. The external identification of PS through Annexin V conjugates was carried out using flow cytometry. Seminal samples containing 5 × 10^6^ spermatozoa per mL were washed and centrifuged (800 × g for 5 min) in HEPES buffer (10 mM HEPES, 140 mM NaCl, 2.5 mM CaCl_2_, pH 7.4). The samples were subsequently resuspended in HEPES buffer, followed by the separation of 5 L of allophycocyanin-annexin V and 0.1 L of 0.005 mM stock solution, after which green nucleic acid-stained DNA dye (Sytox, Molecular Probes, Eugene, OR, USA) was introduced. Each 100 L of sperm cell suspension in this mixture was incubated for 15 min at room temperature, followed by repeated washing and centrifugation steps (800 × g for 5 min). Both positive (apoptosis induction with H_2_O_2_) and negative (HEPES buffer lacking Ca^2+^) controls were included. Finally, 500 L of HEPES buffer was added, and the samples were promptly subjected to flow cytometry analysis. The configuration included 488 and 635 nm excitation with a 30/530 nm bandpass filter for the detection of the fluorescein isothiocyanate (FITC) conjugate (Sytox) and a 16.661 nm bandpass filter for allophycocyanin (Annexin V) [35].* Analysis of Sperm Apoptosis by Flow Cytometry. It should be supported with representative photomicrograph.*

### RT-qPCR

Total RNA was extracted from testicular tissue using TRIzol reagent (Invitrogen, USA). The extracted RNA was subsequently reverse transcribed to generate cDNA using a commercially procurable kit (Thermo Fisher Scientific, Waltham, MA, USA) in conjunction with RT-PCR (Real-time Rotor gene, Germany). The Qrt-PCR was performed in a total volume of 20 μL, comprising 2 μL of cDNA (five times diluted), 0.5 μL of 5 mmol/L solutions of both forward and reverse primers (Table 1), and 10 μL of 2X SYBR DNA PCR. Each sample was subjected to duplicate analyses. The sequences of the forward and reverse primers were as follows. Melt curve analysis was conducted for each iteration to examine the existence of nonspecific PCR products and primer dimers. The expression ratio was determined using the relative formula derived from the comparative CT method (ΔΔCT).

**Table 1:** Primer sequences

Gene	Sequence 5'−3'
*Rat-Nrf2-F*	CAGCACATCCAGACAGACAC
*Rat-Nrf2-R*	AATATCCAGGGCAAGCGACT
*Rat-B2M-F*	CGTCGTGCTTGCCATTCAG
*Rat-B2M-R*	GCAGTTGAGGAAGTTGGGC
*Rat-NF-κB-F*	ACAACTATGAGGTCTCTGGG
*Rat-NF-κB-R*	CTATGTGCTGTCTTGTGGAG
*Rat-Sirt1-F*	TTCAAGGCTGTTGGTTCCAG
*Rat- Sirt1-R*	AGGCGTCATCTTCAGAGTCT
*Rat-FOXO1-F*	ACTTCAAGGATAAGGGCGACA
*Rat- FOXO1-R*	ATTTCCCACTCTTGCCTCCC
*Rat-TGF-β-F*	GACATGAACCGACCCTTCCT
*Rat-TGF-β-R*	GCCGTACACAGCAGTTCTTC

### Statistical analysis

The analysis was conducted using GraphPad Prism software (version 9). Differences among the experimental groups were analyzed using one-way analysis of variance (ANOVA) coupled with Tukey’s post hoc test. The data are presented as mean ± SD. Statistical significance was set at P < 0.05.

## Results

### Measurement of the antioxidant properties of *M.S* leaves

The antioxidant properties of *M.S* leaves were evaluated using the DPPH test method. Based on the findings, the 70% ethanol extract of *M.S* showed an overall efficacy of 42.372% in suppressing DPPH radicals. The antioxidant activity of *M.S* was computed using the formula [(Ac-As) ÷ Ac] × 100, where Ac represents the absorbance of the control and As represents the absorbance of the sample, resulting in an antioxidant activity of 42.372%. Vitamin C was also used as an indicator of antioxidant activity, and its IC_50_ was measured at 2.3 mg, while the IC_50_ of the *M.S* extract was measured at 1.18 mg. The closer the IC_50_ is to that of vitamin C, the greater the antioxidant potential.

### Varicocele induction and testicular histopathology evaluation

**Fig. 1:**
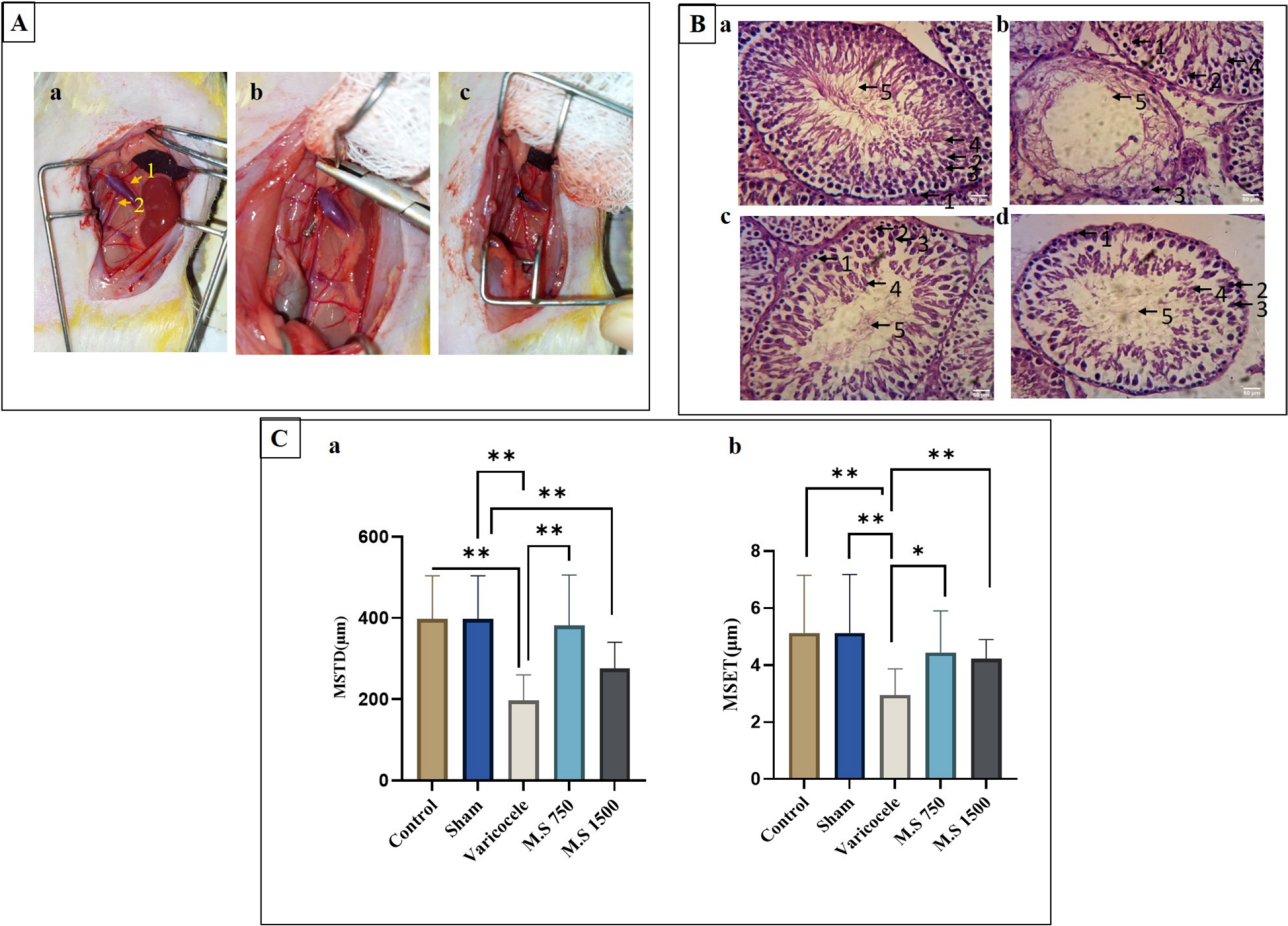
**A** Surgery and induction of the varicocele model in adult male Wistar rats. The left renal vein, from which the internal spermatic vein branches off, was tied. a: Location of the renal vein and internal spermatic vein relative to the kidney, b: needle passage to isolate the renal vein, c: renal vein node. 1: Renal vein, 2: spermatic vein.** B** Histological analysis of the testicular tissue of the studied groups using hematoxylin and eosin staining. a: Control group, b: varicocele group, c: M.S 750 mg/kg treatment group, d: M.S 1500 mg/kg treatment group, 1: Spermatogonial cell, 2: primary spermatocyte cell, 3: secondary spermatocyte cell, 4: spermatid, 5: sperm.** C** Histological analysis of a: mean seminiferous tubule diameter, b: mean seminiferous epithelial thickness. **D** The number of spermatogonia, primary spermatocytes, and spermatids. The extent of destruction in the induction group and repair in the treatment group can be seen. Differences among the experimental groups were analyzed using one-way analysis of variance (ANOVA) coupled with Tukey’s post hoc test. All values are expressed as mean ± SD. A *p* value of ≤ 0.05 was considered significant. *P* < 0.05(*), *P* < 0.01(**), *P* < 0.001(***). M.S: *Malva sylvestris*

The H&E staining revealed that varicocele disrupts the architecture of testicular tissue (Fig. 1A; a, b, c). Compared to the control group, rats in the varicocele model exhibited substantial impairment of testicular spermatogenesis and sperm maturation disorders, resulting in thinner and more irregular seminiferous tubules (Fig. 1B). The mean seminiferous tubule diameter (MSTD) was 397.743 ± 7.6 μm in the control group, whereas in the varicocele group, it was 196.808 ± 13.2 μm (*P* < 0.01). There was also a significant difference in the mean seminiferous epithelial thickness (MSET) between the control group (5.131 ± 2.6 μm) and the varicocele group (2.947 ± 1.3 μm), indicating a reduction in the varicocele model group. Compared to the varicocele model group, treatment with *M.S* showed clear reparative effects, as evidenced by the increased thickness of MSTD and MSET in the spermatogenic epithelium, as well as the presence of a greater number of germ cells and mature sperm (Fig. 1C). Although there was a minor enhancement in grams per kilogram, it did not reach statistical significance. Moreover, the testicular structure did not fully revert to its original state. The results of cell enumeration within 100 spermatogenic tubes are detailed in (Table 2).

**Table 2:** Cell analysis spermatogenic tubes

Cell Type	Control	Sham	Varicocele	M.S 750	M.S 1500
Spermatogonia	58 ± 12	56 ± 02	22 ± 16^***^	59 ± 7^***^	56 ± 9^***^
Primary Spermatocyte	46 ± 10	44 ± 14	13 ± 8^***^	44 ± 12^***^	34 ± 7^***^
Spermatid	97 ± 13	94 ± 18	37 ± 15^***^	76 ± 11^**^	73 ± 6^***^

*M.S Malva sylvestris*. Differences among the experimental groups were analyzed using one-way analysis of variance (ANOVA) coupled with Tukey’s post hoc test. The data are presented as mean ± SD. Statistical significance was determined by *P* < 0.01(**), and *P* < 0.001(***)

### Evaluation of epididymal sperm parameters

**Fig. 2:**
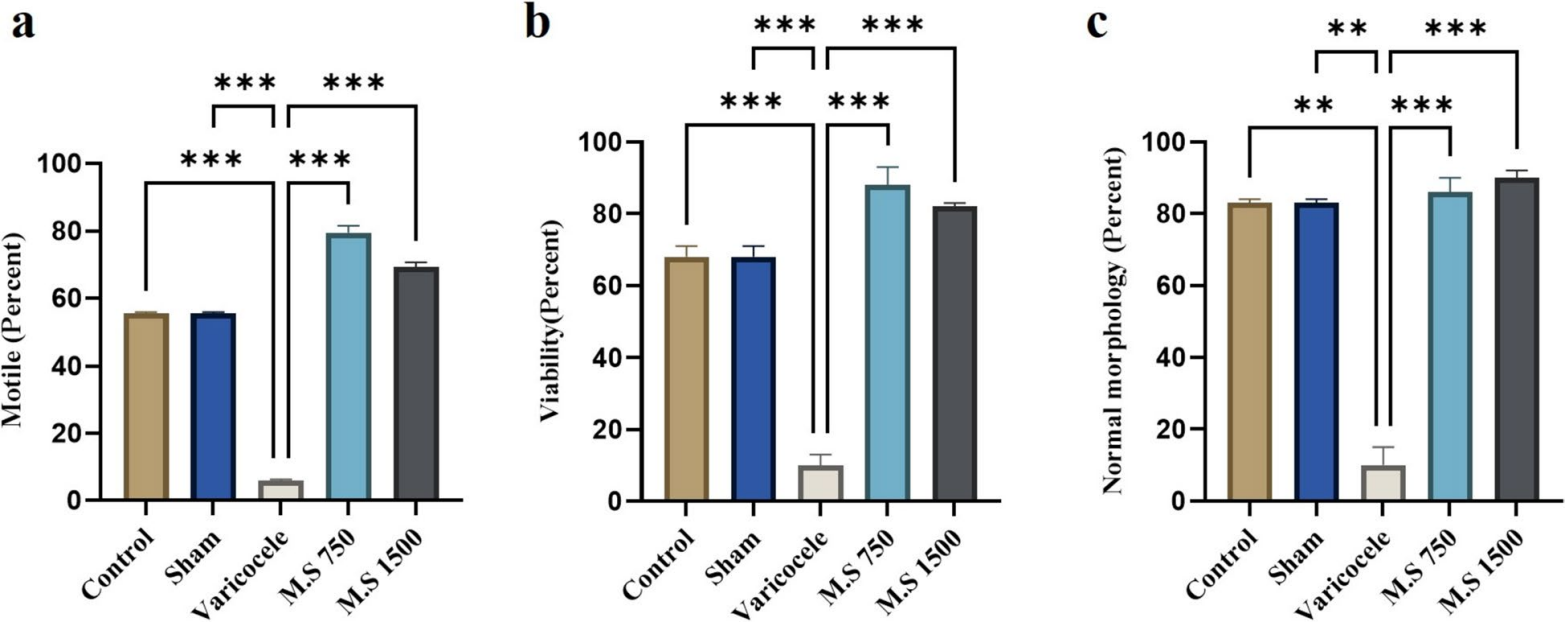
Comparison of epididymal sperm parameters. a: Motility, b: viability, c: normal sperm morphology in the left testicle. The treatment groups showed a significant increase compared to the induction group. M.S 750: M.S 750 mg/kg, M.S 1500: M.S 1500 mg/kg, ns = 8. Differences among the experimental groups were analyzed using one-way analysis of variance (ANOVA) coupled with Tukey’s post hoc test. A *p* value of ≤ 0.05 was considered significant*. P* < 0.05(*), *P* < 0.01(**), *P* < 0.001(***). M.S: *Malva sylvestris*

The results of the CASA investigation, which juxtaposes sperm characteristics across four control sets, varicocele, and treated sets, are illustrated in Figs. 2 and Table 3. The viability, motility, and morphology of the sperm from the left testis were notably lower in the varicocele group than in the control group (*P* < 0.001). However, no significant difference was noted compared to the control group.

**Table 3:** Parameters used in sperm motility analysis

Parameters	Control	Sham	Varicocele	M.S 750	M.S 1500
PR (%)	31.08 ± 0.8	34.78 ± 3.80	11 ± 0.99^***^	51.11 ± 0.5^****^	45.17 ± 0.77^***^
NP (%)	24.19 ± 0.55	23.6 ± 2.05	5.53 ± 0.599^***^	28.36 ± 0.166^***^	24.71 ± 0.21^***^
IM (%)	44.73 ± 3.75	42.01 ± 0.75	94.21 ± 2.23^***^	20.52 ± 2.50^***^	30.12 ± 1.15^***^
M (%)	55.27 ± 0.59	52.32 ± 1.09	5.79 ± 0.30^***^	79.48 ± 2.01^***^	69.88 ± 2.30^***^
VCL(μm/sec)	37.6 ± 2.02	35.6 ± 0.22	1.36 ± 1.03^***^	44.17 ± 1.5^***^	43.17 ± 2.5^***^
VSL (μm/sec)	12.30 ± 1.05	12.27 ± 0.59	0.60 ± 0.53^***^	17.31 ± 1^***^	16.47 ± 0.5^***^
VAP (μm/sec)	14.85 ± 2	15.78 ± 0.789	0.60 ± 0.60^**^	23.13 ± 0.13^***^	20.63 ± 1^***^
LIN (%)	15.68 ± 1.01	16.85 ± 2	3.7 ± 0.7^***^	18.37 ± 0.379^***^	17.54 ± 0.545^***^
STR (%)	36.73 ± 0.739	33.68 ± 1.21	13.33 ± 0.335^***^	38.01 ± 1^***^	32.46 ± 0.46^****^
WOB(%)	33.92 ± 0.92	31.92 ± 0.92	0.70 ± 0.5^***^	44.37 ± 0.37^***^	41.27 ± 0.27^***^
ALH(%)	2.59 ± 0.5	2.09 ± 0.6	0.21 ± 0.2^*^	2.97 ± 0.7^***^	2.87 ± 0.7^***^
BCF(%)	1.45 ± 0.5	1.32 ± 0.4	0.30 ± 0.3^*^	1.90 ± 0.5^**^	1.95 ± 0.5^**^

*M.S* Malva sylvestris, *PR* progressive, *NP* nonprogressive, *IM* immotile, *M* motile, *VCL* velocity of the curved line, *VSL* velocity of straight line, *VAP* velocity of average pathway, *LIN* linearity, *STR* straightness, *WOB* wobble index, *ALH* amplitude lateral head displacement, *BCF* beat frequency. Differences among the experimental groups were analyzed using one-way analysis of variance (ANOVA) coupled with Tukey’s post hoc test. The data are presented as mean ± SD. Statistical significance was determined by *P* < 0.05(*), *P* < 0.01(**), and *P* < 0.001(***)

### DFI sperm chromatin structure

**Fig. 3:**
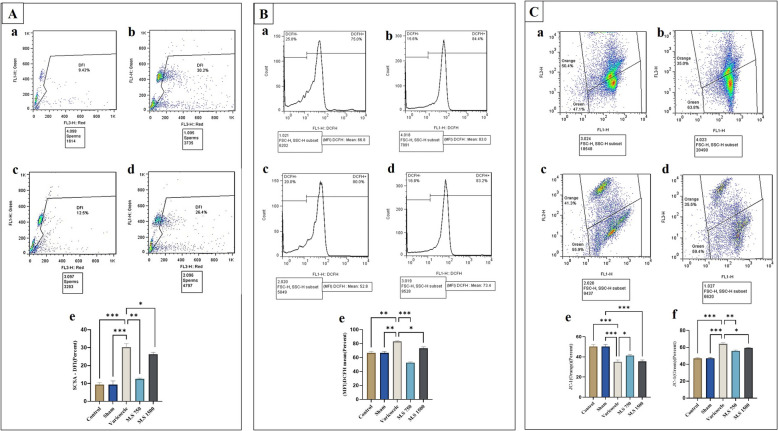
Effects of M.S treatment (750 and 1500 mg/kg) on Sperm Chromatin Structure in varicocele-induced rats. **A** Comparison of sperm DFI strand damage through sperm chromatin structure analysis. **B** Comparison of DFI in the studied groups.** C** Comparison of the level of apoptosis by examining the MMP of the tissue testis with JC-1 dye in a: the control group, b: the varicocele group, c: the 750 mg/kg treatment group, and d: the 1500 mg/kg group. The extent of damage in the induction group and repair in the treatment group can be seen in this image, according to the chromatin structure (DFI) and mitochondrial membrane staining. Differences among the experimental groups were analyzed using one-way analysis of variance (ANOVA) coupled with Tukey’s post hoc test. A *p* value of ≤ 0.05 was considered significant. *P* < 0.05(*), *P* < 0.01(**), *P* < 0.001(***). M.S: *Malva sylvestris*; DFI: DNA Fragmentation Index; SCSA: Sperm Chromatin Structure Assay; DFI: DNA Fragmentation Index

Figure 3A depicts the comparison of the sperm DFI using the SCSA. Significant differences were observed in sperm chromatin following partial occlusion of the left renal vein. There was a significant increase in perm DFI in the varicocele group (*P* < 0.001). In contrast to the model group, the *M.S* 750 mg/kg treatment group exhibited a significant reduction in the epididymal sperm DFI (*P* < 0.01).

### ROS

Varicocele results in the disruption of local oxidative equilibrium, leading to the initiation of OS and the overproduction of ROS. Oxidative stress in turn reduces and perturbs the total antioxidant capacity (TAC) of the overall seminal plasma and sperm parameters [36]. Compared to the control group, ROS levels significantly increased in rats induced with bilateral varicocele (*P* < 0.01). Compared to the varicocele model group, the average DCFH levels of epididymal sperm were significantly lower in the *M.S* 750 mg/kg intervention group (*P* < 0.001), as depicted in Fig. 3B.

### JC-1

The evaluation of the MMP serves as a more accurate indicator of the performance of Mitochondria [33]. Compared to the control group, the varicocele group demonstrated the highest level of apoptosis in terms of MMP (*P* < 0.001). Moreover, the extent of MMP damage was slightly greater in the 1500 mg/kg treatment group than in the 750 mg/kg treatment group. Interestingly, the orange fluorescence associated with varicocele was significantly more pronounced in the 750 mg/kg treatment group (*P* < 0.001), suggesting a reduction in apoptosis within this group (Fig. 3C).

### Apoptosis

**Fig. 4:**
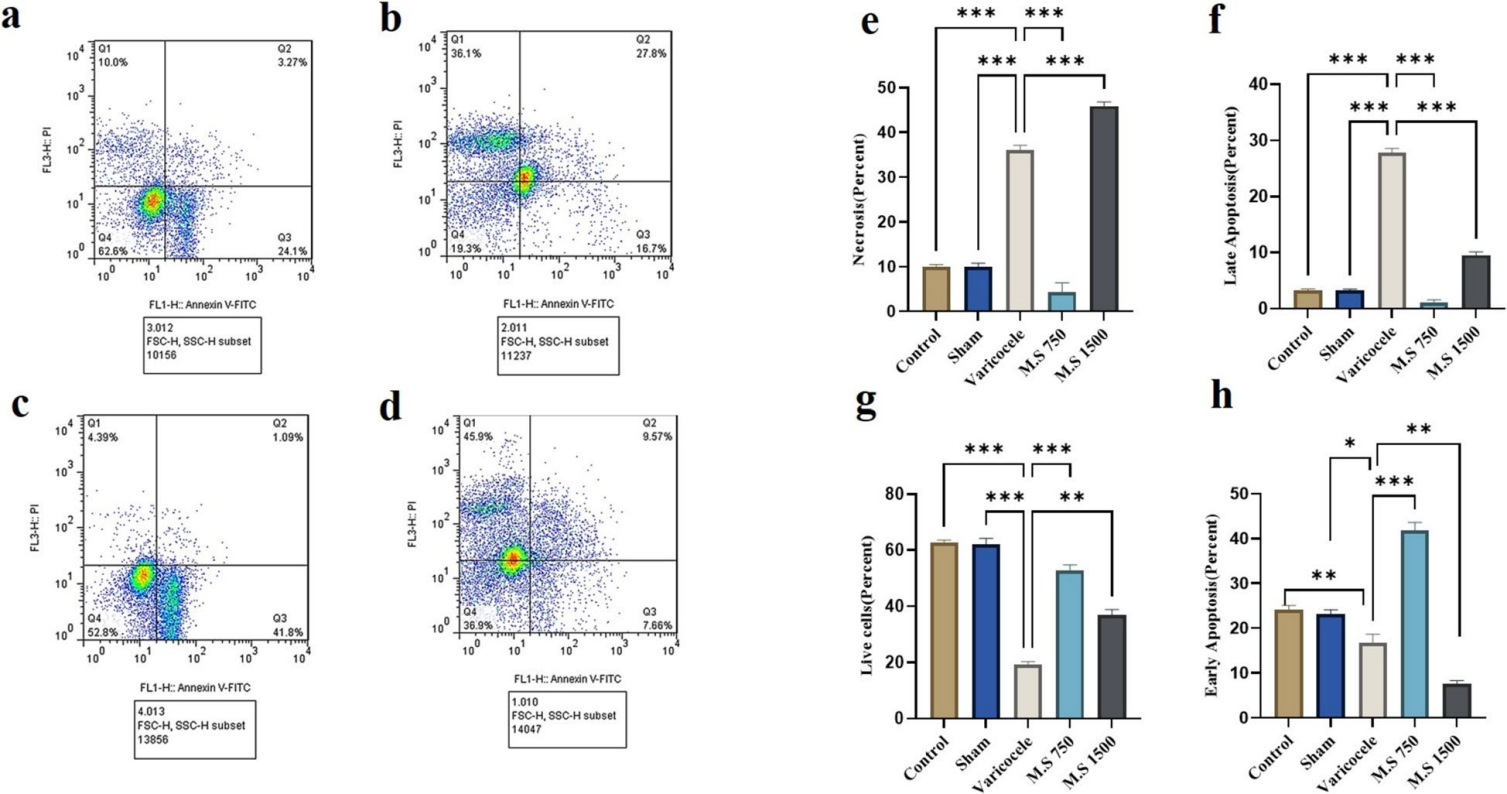
Comparison of sperm cell apoptosis in the investigated groups. **a** Control group, **b** varicocele group, **c** 750 mg/kg treatment group, **d** 1500 mg/kg treatment group, **e** comparison of sperm cell necrosis in the investigated groups, **f** comparison of secondary apoptosis in sperm cells in the studied groups, **g** comparison of living sperm cells in the studied groups, **h** comparison of primary apoptosis in sperm cells in the studied groups. Differences in live and apoptotic cells are observed in the induction and treatment groups using one-way analysis of variance (ANOVA) coupled with Tukey’s post hoc test. A *p* value of ≤ 0.05 was considered significant*. P* < 0.05(*), *P* < 0.01(**), *P* < 0.001(***). M.S 750: M.S 750 mg/kg, M.S 1500: M.S 1500 mg/kg. Q1: Necrosis, Q2: Late apoptosis, Q3: Early apoptosis, Q4: Live cells. M.S: *Malva sylvestris*

Apoptosis usually occurs during normal spermatogenesis, resulting in the loss of 75% of the potential sperm count. As a result, only 25% of the primary spermatocytes expected from A spermatogonia are produced. Disruption of apoptosis at this stage results in a male infertility phenotype. A comparison was made between varicocele rats and those receiving treatment with *M.S*. The proportion of sperm cells exhibiting phosphatidylserine displacement, which was located in the lower right quadrant and denoting primary apoptosis, was significantly greater in the varicocele model than in the treatment groups (Fig. 4).

### RT-qPCR

**Fig. 5:**
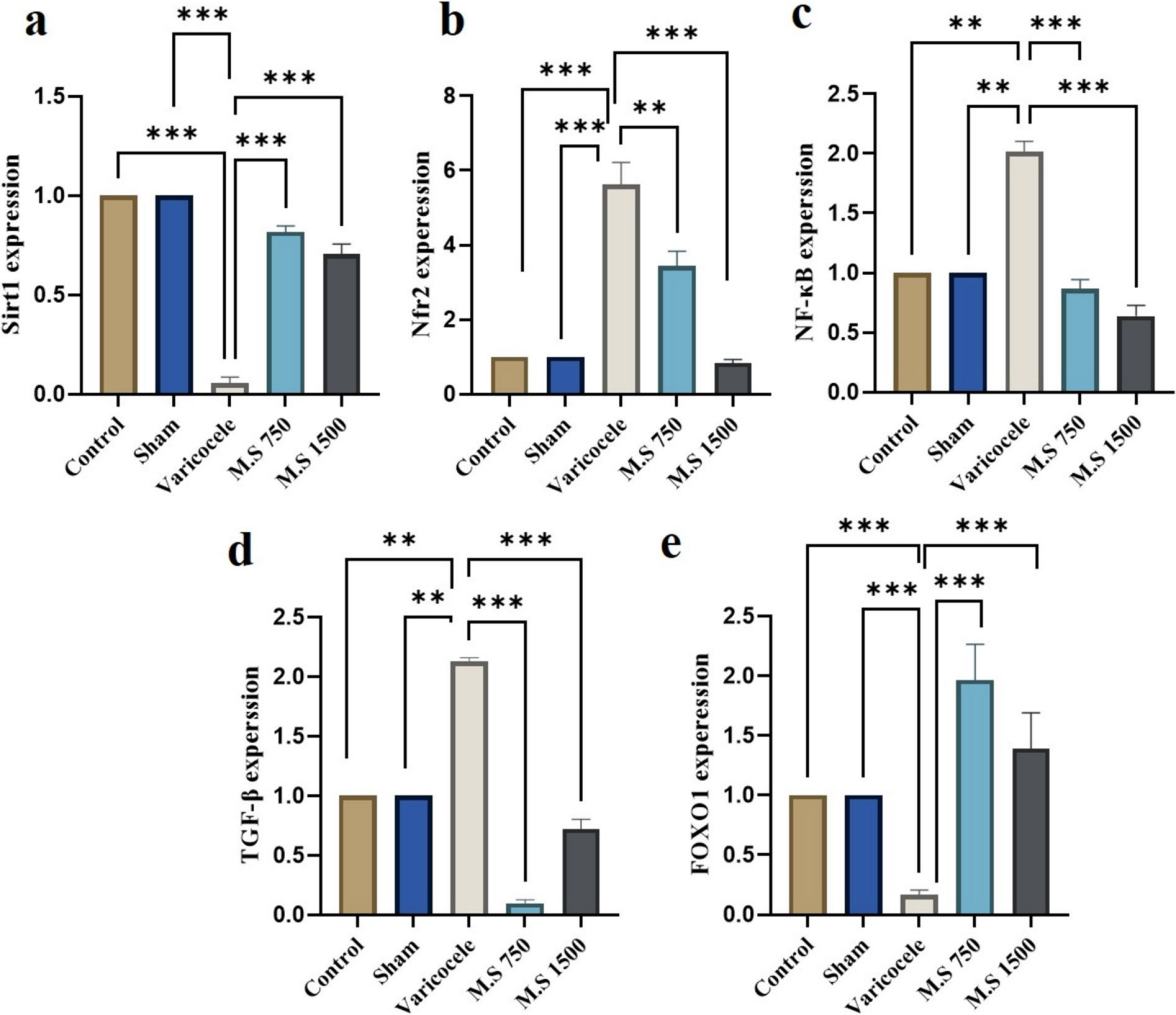
Effects of M.S treatment (750 and 1500 mg/kg) on SIRT1, FOXO1, NF-κB, and TGF-β gene expression in the varicocele model. **a** SIRT1 gene expression, **b** NRF2 gene expression, **c** NF-κB gene expression, **d** TGF-β gene expression, **e** FOXO1 gene expression. *Malva Sylvestris* shows increased expression of SIRT1 and FOX01 genes and decreased expression of NF-kB, TGF-β, and NRF2 genes. Differences among the experimental groups were analyzed using one-way analysis of variance (ANOVA) coupled with Tukey’s post hoc test. A *p* value of ≤ 0.05 was considered significant*. P* < 0.05(*), *P* < 0.01(**), *P* < 0.001(***). M.S: *Malva sylvestris*

To investigate how epigenetic involvement could have potentially modulated the impact of *M.S* at a dose of 750 mg/kg in the testicular tissues of the varicocele model, the expression levels of *SIRT1*, *FOXO1*, *NRF2*, *NF-κB*, and *TGF-β* genes were assessed. The enzymatic activities of *SIRT1*, *FOXO1*, and *TGF-β* were significantly reduced in the testes following the development of experimental varicocele. However, after the 21-day administration of *M.S*, this trend was reversed (Fig. 5). *NF-κB* gene expression was upregulated in the varicocele group compared to the control group which experienced no significant changes following subsequent treatment with *M.S*. Furthermore, *NRF2* gene expression was significantly lower in the varicocele group than in the control group (*P* < 0.05) while in the treatment group, it was significantly lower than in the varicocele group (*P* < 0.001).

## Discussion

This study underscores the complex interplay between oxidative stress (OS), genetic regulation, and apoptosis in varicocele-induced infertility, while highlighting the therapeutic potential of *M.S* extract in mitigating these effects. The findings demonstrate that *M.S* administration, particularly at 750 mg/kg, significantly improves sperm quality, reduces DNA fragmentation, and restores mitochondrial membrane potential by modulating key antioxidant and anti-inflammatory pathways. Its efficacy is linked to the upregulation of SIRT1 and FOXO1, which enhance cellular repair and antioxidant defenses, and the suppression of NF-κB, TGF-β, and NRF2, thereby attenuating OS and inflammation.

In comparison, the synthetic antioxidants butylated hydroxyl toluene (BHT) and ascorbic acid presented significantly higher FRAP values of 0.715 and 1.854, respectively, at the same concentration. Although the half-maximal effective concentration (EC50) value of the *M.S* flower extract at 21.52 μg/mL was higher than the positive controls BHT and α-tocopherol at 3.23 and 2.25 μg/mL, respectively, it showed significant antioxidant properties by inhibiting active oxygen [37]. *M.S* exhibited an antioxidant activity of 42.372% and a DPPH half-maximal inhibitory concentration (IC_50_) of 1.180. Ben Saad et al. (2017) investigated the impact of *M.S* extract on mitigating the detrimental effects of lithium carbonate on the testicles and heart. Their findings indicated that *M.S* effectively mitigates the harmful effects of lithium carbonate, which can be attributed to its interaction with intricate polysaccharides possessing antioxidative characteristics [22].

Recent studies have proposed that conditions such as ion imbalance, hypoxia, and alterations in blood circulation have the potential to elevate ROS in varicocele patients. ROS attack polyunsaturated fatty acids in sperm membranes, reducing motility and membrane integrity. ROS induce DNA strand breaks, leading to genetic abnormalities and reduced fertilization potential. Excessive ROS impair ATP production, essential for sperm motility. Antioxidants neutralize ROS, restore redox balance, and protect sperm integrity [38]. In the clinical setting, antioxidant compounds, such as kallikrein, L-carnitine, anthocyanin, silymarin, chrysin, selenium, resveratrol, and other similar agents are utilized for the management of varicocele. [39]. A clinical trial on 300 sub-fertile males showed that antioxidant blends significantly improved sperm count, motility, and reduced DFI in severe oligospermia cases [40, 41]. Examinations of various components of *M.S* have revealed that the leaves exhibit greater efficacy than the petiole. This disparity is attributed to flavonoid compounds with robust antioxidant properties. *M.S* flower extract has also displayed major antioxidant capabilities, attributed to various compounds found within this botanical sample, such as phenols, flavonoids, and tocopherols [20, 37]. The assessment of the antioxidant potential of the methanolic extract of *M.S* flowers revealed a reduction of 0.355 at 0.5 mg/mL.

The present study examined the impact of *M.S* on sperm quality within a varicocele model. H&E staining, sperm count, and motility analyses indicated that *M.S* enhances spermatogenesis and mitigates testicular tissue injury. Sperm DFI serves as a quantitative measure for assessing sperm DNA damage, which is a significant contributor to male infertility. The increase in ROS levels can disrupt the inflammatory milieu, potentially raising DFI and impairing mitochondrial function and motility, which in turn leads to reproductive dysfunction [42, 43]. In the current study, sperm DFI was higher in the varicocele model group than in the control group, which weakened sperm motility. Following a 21-day treatment with *M.S*, a significant reduction was observed in sperm DFI.

Mitochondria play a key role in the production of ROS and superoxide radicals under low oxygen, thereby affecting sperm quality. The hypoxic-ischemic shock in varicocele can change sperm mitochondria, which leads to abnormal sperm and infertility. In addition, hyperpolarization of the sperm MMP increases superoxide production [33, 44]. In the present study, the relationship between mobility and MMP was evident. While there was a decline in mobility in the varicocele group compared to the control group, a marked improvement was observed in the mobility of *M.S* treatment groups compared to the varicocele group. Research has suggested that antioxidants facilitate the recovery of MMP and sperm motility. The results of the DPPH analysis showed that the level of antioxidants in *M.S* was satisfactory. Therefore, the antioxidant attributes of *M.S* can contribute to the restoration of MMP [35]. This study also found that ROS levels increase in varicocele, as increased thermal stress is associated with increased generation of ROS by cellular organelles, such as mitochondria, the plasma membrane, the cytoplasm, and peroxisomes [45].

The bioactive compounds in *Malva salvistris*—including flavonoids, polyphenols, and terpenoids—appear to directly inhibit NADPH oxidase (NOX enzymes), a major contributor to reactive oxygen species (ROS) generation in varicocele. Flavonoids such as quercetin and kaempferol may bind to critical NOX subunits, disrupting enzyme assembly and activity, thereby suppressing NOX-driven oxidative stress. By blocking NADPH oxidase, *Malva* diminishes the production of superoxide (O₂⁻) and other ROS in testicular and sperm cells [15, 20]. Additionally, its phytochemicals act as direct ROS scavengers and enhance endogenous antioxidant defenses, such as superoxide dismutase (SOD) and glutathione, a mechanism supported by studies demonstrating *Malva*’s ROS-lowering effects in diabetic and inflammatory models [18, 46]. The reduction in ROS alleviates oxidative damage to mitochondrial membranes, preventing cytochrome c release and subsequent activation of caspase-9, which in turn suppresses downstream caspase-3 activity. This is critical, as elevated caspase-3 levels are strongly associated with sperm apoptosis in varicocele patients.Ultimately, the inhibition of caspase-3 helps preserve sperm DNA integrity and cellular viability, offering a protective mechanism against varicocele-induced infertility [47–49]. In this study, compared to the control group, the varicocele group presented diminished counts of viable cells and cells undergoing primary apoptosis. On the other hand, there was a significant increase in necrosis and secondary apoptosis levels in the varicocele group, indicating an advanced progression of apoptosis within the sperm cells, ultimately leading to necrosis. In the *M.S* 750 mg/kg treatment group, more viable cells and sperm cells undergoing primary apoptosis were detected than in the varicocele group. There was also a significant decline in the number of sperm cells undergoing secondary apoptosis and necrosis, which indicates the beneficial effects of *M.S* hydroalcoholic extract on spermatogenesis, and suggests that this extract, which functions as an antioxidant, possibly impedes the transition of sperm cells into subsequent apoptosis phases. However, the *M.S* 1500 mg/kg treatment group, compared to the varicocele group, presented notable increases in necrosis levels. There was also a significant decline in primary and secondary apoptosis in this particular group, signifying the progression of sperm cells toward necrosis. Therefore, this concentration of the extract did not yield optimal outcomes in managing the apoptosis process in varicocele pathology and potentially exerted cytotoxic effects.

The regulatory gene responsible for silencing information, known as *SIRT1*, plays a crucial role in enhancing the production of antioxidants, facilitating the repair of cells that have been compromised due to OS, and preventing cellular malfunction. Depletion of *SIRT1* results in impaired mitochondrial function accompanied by elevated levels of ROS, increased lipid peroxidation, and DNA harm in both male and female reproductive cells (sperm and eggs), ultimately causing infertility [50]. Matthew Coussens et al. (2008) revealed that spermatogenesis is significantly reduced as a result of *SIRT1* deficiency [51]. In another study, Taymour Mostafa et al. (2020) assessed *SIRT1* expression levels in males diagnosed with varicocele following surgical intervention, revealing a significant reduction in *SIRT1* in sperm, subsequently leading to improvements in sperm parameters. Furthermore, this phenomenon was found to be associated with changes in seminal fluid composition [52]. Mostafa et al. reported that OS and *SIRT1* deficiency are the causes of male infertility in varicocele patients. Varicocele also induces OS, and *SIRT1* deficiency intensifies the effect of reducing the antioxidant defense of sperm [53, 54].

The *FOXO1* gene belongs to the FOXO protein family, which regulates the advancement of spermatogenesis, starting from the sustained self-renewal of spermatogonia to the onset of spermatogenesis and meiosis [55]. The reduction in *SIRT1*/*FOXO1* testicular expression levels in the varicocele group suggests a correlation between varicocele-related fertility disorders and the *SIRT1*/*FOXO1*/OS axis. The upregulation of *SIRT1* activity modulated by *M.S* in the experimental group implies a potential molecular interplay between *M.S* and *SIRT1* in varicocele-triggered OS, accompanied by increased *FOXO1* expression and antioxidant capabilities. The ability of *M.S* to increase *FOXO1* levels may be attributed to *SIRT1*-mediated *FOXO1* deacetylation, which facilitates the nuclear translocation, retention, and transcriptional function of its antioxidant enzymes. *SIRT1*-mediated deacetylation of proliferator-activated receptor gamma coactivator 1-alpha (PPARGC1A) enhances mitochondrial biogenesis and metabolic oxidative process while suppressing proinflammatory pathways. Furthermore, nuclear *SIRT1* expression has a role in the modulation of nuclear factor erythroid 2-related factor 2 (*NRF2*) expression and the stimulation of robust transcription of antioxidant enzymes [19]. Yong Wang et al. (2018) revealed that grape seed proanthocyanidin extract (GSPE) can alleviate testicular oxidative damage resulting from varicocele by activating nuclear (erythroid-derived 2) antioxidant pathway (*NRF2*)[56].

*NRF2* serves as a transcription factor responsible for upregulating the expression of antioxidant proteins, thereby exerting protective effects against OS-induced damage [57]. *SIRT1*, in conjunction with *NF-κB*, represents an additional prospective pathway that serves a significant function in diverse biological phenomena, such as inflammation, apoptosis, and OS [58]. Volkan Tuğcu et al. (2010) reported the *NF-κB* transcription factor in varicocele experimental models [59]. A link has been found between the reduced expression of *SIRT1* in the varicocele group and elevated levels of acetylated *NF-κB*, which can be attributed to compromised mitochondrial biogenesis, heightened OS, and increased inflammatory responses. Furthermore, the dysfunction of mitochondria, which leads to the disruption of MMP, has been found to strongly associate with irreversible alterations in permeability [60]. These alterations ultimately trigger a series of breakdown reactions that result in increased apoptotic cell death capacity, as revealed in the present study.

Previous studies have also shown that transforming growth factor-beta (TGF-β) levels are elevated in individuals diagnosed with varicocele, indicating a possible link to diminished sperm characteristics and impaired spermatogenesis. A study conducted in 2008 examined epidermal growth factor (EGF) and TGF-β levels in infertile varicocele patients compared to fertile individuals to analyze the association between TGF-β and sperm plasma. The results indicated while the levels of EGF and TGF-Semen plasma β increased in varicocele patients, seminal parameters decreased in this group [61]. Dobashi et al. also found increased expression of TGF-β1 in varicocele testes, indicating TGF-β1 plays a role in seminiferous tubule fibrosis and potentially leads to spermatogenesis impairment [62]. A 2010 study further investigated TGF-β1 expression in the pathogenesis of human testicular tissue. Monohistochemical studies have revealed the presence of TGF-β1 and its specific receptors in Leydig cells in samples obtained from both normal tissue and from patients diagnosed with Sertoli cell-only syndrome (SCO) or hypospermatogenesis. Although the presence of Leydig cell hyperplasia (LCH) is not confirmed, it is suggested that its expression is likely to be elevated [63]. In another study, Kowalewski et al. reported higher TGF-β levels in the varicocele group compared to the control group [64].

The present study revealed a significant reduction in the expression of *SIRT1* and *FOXO1* genes in the varicocele group, demonstrating an association with the level of ROS. Furthermore, there was a significant increase in *NF-κB*, *TGF-β*, and *NRF2* genes in the varicocele group. Previous research has indicated that antioxidative agents confer protection to testicular tissues from OS and apoptosis in varicocele-induced rat models [65, 66]. In essence, this study demonstrated notable alterations in *SIRT1*, *FOXO1*, *NRF2*, *NF-κB*, and *TGF-β* gene expression, and in OS levels in rats induced by varicocele, which aligns with the findings of previous studies. The present findings indicated the cytoprotective capacity of *M.S* at 750 and 1500 mg/kg concentrations as a potent antioxidative agent in ameliorating varicocele-related complications through modulating OS and apoptotic pathways. *M.S* exhibited anti-inflammatory properties by upregulating *SIRT1* and *FOX01* genes while downregulating *NF-κB*, *TGF-β*, and *NRF2* genes, which consequently diminished ROS levels.

The findings from this study and related research position M.S as a promising adjunct therapy for managing OS-driven male infertility, particularly in conditions like varicocele. M.S offers a natural polyphenol-rich alternative with synergistic compounds that enhance ROS scavenging and mitochondrial protection. Post-Varicocelectomy surgical administration of M.S could mitigate residual OS, accelerate recovery of spermatogenesis, and reduce sperm DFI, potentially improving pregnancy rates. While further clinical validation is essential, its ability to modulate SIRT1/FOXO1, inhibit NADPH oxidase, and suppress apoptosis positions it as a compelling candidate for integrative fertility care.

The current study has some limitations. This investigation offers more substantial evidence regarding the antioxidant properties of M.S by analyzing alterations in the expression of proteins that play a role in cellular signaling pathways. Nevertheless, the research team encountered obstacles in acquiring the necessary antibodies because of financial and political limitations. Furthermore, the absence of advancements in functional organoid technology and testicular tissue constructs compelled the team to depend on animal models for this study.

## Conclusions

The findings of this study, along with previous research, imply that *M.S* extract can alleviate tissue damage caused by OS due to varicocele in the testicular region. These findings provide fundamental insights into the positive effects of *M.S* extract on sperm characteristics in male rats with experimental varicocele. The findings also show that *M.S* substantially contributes to the management of OS, cell viability, and the enhancement of protective mechanisms on testicular tissue in male rats suffering from varicocele. Further studies are needed to ascertain whether *M.S* can improve fertility outcomes in varicocele patients.

## Data Availability

No datasets were generated or analysed during the current study.

## Abbreviations

ROS: Reactive Oxygen Species
OS: Oxidative Stress
CASA: Computer-Assisted Sperm Analysis
VCL: Curvilinear Velocity
VAP: Average Path Velocity
VSL: Straight-Line Velocity
LIN: Linearity Index
STR: Oscillation Index
WOB: Wobbling Index
BCF: Beat Cross Frequency
ALH: Amplitude of Lateral Head
H&E: Hematoxylin And Eosin
PBS: Phosphate-Buffered Saline
SCSA: Sperm Chromatin Structure Assay
DFI: DNA Fragmentation Index
HDS: High-Density Staining
DCFH-DA: 2',7'-Dichlorofluorescein Diacetate
H2O2: Hydrogen Peroxide
DHE: Dihydroethidium
DCF: Dichlorofluorescein
HE: 2-Hydroxyethidium
MMP: Mitochondrial Membrane Potential
PS: Phosphatidylserine
FITC: Fluorescein Isothiocyanate
one-way ANOVA: One-way Analysis of Variance
MSTD: Mean Seminiferous Tubule Diameter
MSET: Mean Seminiferous Epithelial Thickness
TAC: Total Antioxidant Capacity
BHT: Butylated Hydroxyl Toluene
IC_50_: Half-maximal Inhibitory Concentration
EC50: Half-maximal Effective Concentration
PPARGC1A: Proliferator-Activated Receptor Gamma Coactivator 1-Alpha
NRF2: Nuclear Factor Erythroid 2-Related Factor 2
EGF: Epidermal Growth Factor
TGF-β: Transforming Growth Factor-Beta
LCH: Leydig Cell Hyperplasia
SCO: Sertoli Cell-Only syndrome

## References

[1] Babakhanzadeh E, Nazari M, Ghasemifar S, Khodadadian A. Some of the factors involved in male infertility: a prospective review. Int J Gen Med. 2020;13:29–41. Available from: https://www.tandfonline.com/action/journalInformation?journalCode=dijg20. Cited 2023 Jun 7. 32104049 10.2147/IJGM.S241099 PMC7008178

[2] Alharbi M, Zini A. Epidemiology of varicocele in pediatric, adolescent, and adult populations. Varicoc Male Infertil. 2019;70:97–106. Available from: https://link.springer.com/chapter/10.1007/978-3-319-79102-9_8. Cited 2023 Jun 9.

[3] Moein MR, Soleimani M, Tabibnejad N. Reactive oxygen species (ROS) production in seminal fluid correlate with the severity of vericocele in infertile men. Iranian J Reprod Med. 2008;6:15.

[4] Rao L, Babu A, Kanakavalli M, Padmalatha V, Singh A, Singh PK, et al. Chromosomal abnormalities and Y chromosome microdeletions in infertile men with varicocele and idiopathic infertility of South Indian Origin. J Androl. 2004;25:147–53. 14662798 10.1002/j.1939-4640.2004.tb02770.x

[5] Wang K, Gao Y, Wang C, Liang M, Liao Y, Hu K. Role of oxidative stress in varicocele. Front Genet. 2022;13:850114. 35401656 10.3389/fgene.2022.850114 PMC8984266

[6] Baskaran S, Finelli R, Agarwal A, Henkel R. Reactive oxygen species in male reproduction: a boon or a bane? Andrologia. 2021;53:e13577. 32271474 10.1111/and.13577

[7] Su JS, Farber NJ, Vij SC. Pathophysiology and treatment options of varicocele: An overview. Andrologia. 2021;53:e13576. 32271477 10.1111/and.13576

[8] Shokoohi M, Khaki AA, Roshangar L, Nasr Esfahani MH, Soltani GG, Alihemmati A. The impact of N-acetylcysteine on hypoxia-induced testicular apoptosis in male rats: TUNEL and IHC findings. Heliyon. 2024;10:e40097. 39748984 10.1016/j.heliyon.2024.e40097 PMC11693919

[9] Parsania S, Salehpoor S, SadrrAmeli M, Abdi M, Shokoohi M, RahimiMamaghani A, et al. Investigating the therapeutic potential of clove extract in mitigating testicular injury associated with testicular hypoxia in male rats. Crescent J Med Biolog Sci. 2024;11:128–34.

[10] Abadi ARR, Boukani LM, Shokoohi M, Vaezi N, Mahmoodi M, Gharekhani M, et al. The flavonoid chrysin protects against testicular apoptosis induced by torsion/detorsion in adult rats. Roy VK, editor. Andrologia. 2023;2023:1–12.

[11] Silay MS, Hoen L, Quadackaers J, Undre S, Bogaert G, Dogan HS, et al. Treatment of varicocele in children and adolescents: a systematic review and meta-analysis from the European association of urology/european society for paediatric urology guidelines panel. Eur Urol. 2019;75:448–61. Available from: https://linkinghub.elsevier.com/retrieve/pii/S0302283818307310. 30316583 10.1016/j.eururo.2018.09.042

[12] Schlegel PN, Sigman M, Collura B, De Jonge CJ, Eisenberg ML, Lamb DJ, et al. Diagnosis and treatment of infertility in men: AUA/ASRM Guideline PART II. J Urol. 2021;205:44–51. Available from: http://www.auajournals.org/doi/10.1097/JU.0000000000001520. 33295258 10.1097/JU.0000000000001520

[13] Hughes EG, Grantmyre J, Zini A. An integrated approach to male-factor subfertility: bridging the gap between fertility specialists trained in urology and gynaecology. J Obstet Gynaecol Canada. 2015;37:258–65. Available from: https://linkinghub.elsevier.com/retrieve/pii/S1701216315303121. 10.1016/S1701-2163(15)30312-1 26001873

[14] Garg H, Kumar R. An update on the role of medical treatment including antioxidant therapy in varicocele. Asian J Androl. 2016;18:222. Available from: https://journals.lww.com/10.4103/1008-682X.171657. 26763549 10.4103/1008-682X.171657 PMC4770490

[15] Batiha GES, Tene ST, Teibo JO, Shaheen HM, Oluwatoba OS, Teibo TKA, et al. The phytochemical profiling, pharmacological activities, and safety of malva sylvestris: a review. Naunyn-Schmiedeberg’s Arch Pharmacol. 2023;396:421–40. 36418467 10.1007/s00210-022-02329-w PMC9898411

[16] Marouane W, Soussi A, Murat JC, Bezzine S, El Feki A. The protective effect of Malva sylvestris on rat kidney damaged by vanadium. Lipids Health Dis. 2011;10:1–8. 10.1186/1476-511X-10-65 PMC3104358 21513564

[17] Iside C, Scafuro M, Nebbioso A, Altucci L. SIRT1 activation by natural phytochemicals: an overview. Front Pharmacol. 2020;11:1225. Available from: https://www.frontiersin.org/article/10.3389/fphar.2020.01225/full. 32848804 10.3389/fphar.2020.01225 PMC7426493

[18] Benso B, Franchin M, Massarioli AP, Paschoal JAR, Alencar SM, Franco GCN, et al. Anti-Inflammatory, Anti-Osteoclastogenic and Antioxidant Effects of Malva sylvestris Extract and Fractions: In Vitro and In Vivo Studies. Bach H, editor. PLOS ONE. 2016;11:e0162728. Available from: https://dx.plos.org/10.1371/journal.pone.0162728. 10.1371/journal.pone.0162728 PMC5028055 27643502

[19] Yang D, Tan X, Lv Z, Liu B, Baiyun R, Lu J, et al. Regulation of Sirt1/Nrf2/TNF-$α$ signaling pathway by luteolin is critical to attenuate acute mercuric chloride exposure induced hepatotoxicity. Sci Rep. 2016;6:37157. Available from: https://www.nature.com/articles/srep37157. 27853236 10.1038/srep37157 PMC5112569

[20] Barros L, Carvalho AM, Ferreira ICFR. Leaves, flowers, immature fruits and leafy flowered stems of Malva sylvestris: a comparative study of the nutraceutical potential and composition. Food Chem Toxicol. 2010;48:1466–72. Available from: https://linkinghub.elsevier.com/retrieve/pii/S0278691510001614. 20233600 10.1016/j.fct.2010.03.012

[21] Alsarayreh AZ, Oran SA, Shakhanbeh JM, Khleifat KM, Al Qaisi YT, Alfarrayeh II, et al. Efficacy of methanolic extracts of some medicinal plants on wound healing in diabetic rats. Heliyon. 2022;8:e10071. Available from: https://linkinghub.elsevier.com/retrieve/pii/S2405844022013597. 35965986 10.1016/j.heliyon.2022.e10071 PMC9364101

[22] Saad AB, Rjeibi I, Alimi H, Ncib S, Smida A, Zouari N, et al. Lithium induced, oxidative stress and related damages in testes and heart in male rats: The protective effects of Malva sylvestris extract. Biomed Pharmacother. 2017;86:127–35. Available from: https://linkinghub.elsevier.com/retrieve/pii/S0753332216321746. 27951419 10.1016/j.biopha.2016.12.004

[23] Mohamadi YZ, Godini A, Madani SH, Najafi H. Reduction of cisplatin-induced renal and hepatic side effects in rat through antioxidative and anti-inflammatory properties of Malva sylvestris L. extract. Biomed Pharmacother. 2018;106:1767–74. Available from: https://linkinghub.elsevier.com/retrieve/pii/S0753332218339416. 30119252 10.1016/j.biopha.2018.07.115

[24] Turner TT. The study of varicocele through the use of animal models. Human reproduction update. Human Reprod Update. 2001;7:78–84. Available from: https://academic.oup.com/humupd/article-lookup/doi/10.1093/humupd/7.1.78. 10.1093/humupd/7.1.78 11212079

[25] Dolatkhah MA, Shokoohi M, Charvandeh S, Tvrda E, Shoorei H, Moghimian M, et al. Fumaria parviflora regulates oxidative stress and apoptosis gene expression in the rat model of varicocele induction. Andrologia. 2020;52:e13826. Available from: https://onlinelibrary.wiley.com/doi/10.1111/and.13826. 32991040 10.1111/and.13826

[26] Alabi TD, de Villiers C, du Plessis SS, Monsees TK, Brooks NL, Oguntibeju OO. The beneficial role of anchomanes difformis in stz-induced reproductive dysfunction in male wistar rats. Diab Metab Syndrome Obes. 2020;13:4543–60. Available from: https://www.dovepress.com/the-beneficial-role-of-anchomanes-difformis-in-stz-induced-reproductiv-peer-reviewed-article-DMSO. 10.2147/DMSO.S270783 PMC7698316 33262627

[27] Sziva RE, Ács J, Tőkés A-M, Korsós-Novák Á, Nádasy GL, Ács N, et al. Accurate quantitative histomorphometric-mathematical image analysis methodology of rodent testicular tissue and its possible future research perspectives in andrology and reproductive medicine. Life. 2022;12:189. Available from: https://www.mdpi.com/2075-1729/12/2/189. 35207477 10.3390/life12020189 PMC8875546

[28] Evenson D, Wixon R. Environmental toxicants cause sperm DNA fragmentation as detected by the Sperm Chromatin Structure Assay (SCSA). Toxicology and Applied Pharmacology. 2005;207:532–7. Available from: https://linkinghub.elsevier.com/retrieve/pii/S0041008X05002413. 10.1016/j.taap.2005.03.021 15987647

[29] Hsu PC, Jhong JY, Huang LP, Lee KH, Chen HP, Guo YL. Transgenerational Effects of Di(2-Ethylhexyl) phthalate on anogenital distance, sperm functions and DNA methylation in rat offspring. Int J Mol Sci. 2021;22:4131. Available from: https://www.mdpi.com/1422-0067/22/8/4131. 33923623 10.3390/ijms22084131 PMC8073582

[30] Evenson DP. The Sperm Chromatin Structure Assay (SCSA®) and other sperm DNA fragmentation tests for evaluation of sperm nuclear DNA integrity as related to fertility. Animal Reproduction Science. 2016;169:56–75. Available from: https://linkinghub.elsevier.com/retrieve/pii/S0378432016300276. 10.1016/j.anireprosci.2016.01.017 26919909

[31] Stahl PJ, Cogan C, Mehta A, Bolyakov A, Paduch DA, Goldstein M. Concordance among sperm deoxyribonucleic acid integrity assays and semen parameters. Fertil Steril. 2015;104:56-61.e1. Available from: https://linkinghub.elsevier.com/retrieve/pii/S0015028215002964. 25989978 10.1016/j.fertnstert.2015.04.023

[32] Fatemi N, Sanati MH, JamaliZavarehei M, Ayat H, Esmaeili V, Golkar-Narenji A, et al. Effect of tertiary-butyl hydroperoxide (TBHP)-induced oxidative stress on mice sperm quality and testis histopathology. Andrologia. 2013;45:232–9. Available from: https://onlinelibrary.wiley.com/doi/10.1111/j.1439-0272.2012.01335.x. 22803951 10.1111/j.1439-0272.2012.01335.x

[33] Alamo A, De Luca C, Mongioì LM, Barbagallo F, Cannarella R, La Vignera S, et al. Mitochondrial membrane potential predicts 4-hour sperm motility. Biomedicines. 2020;8:196. Available from: https://www.mdpi.com/2227-9059/8/7/196. 32645820 10.3390/biomedicines8070196 PMC7400390

[34] De Biasi S, Gibellini L, Cossarizza A. JC-1 Dye for Mitochondrial Membrane Potential - NL. Current Protocols in Cytometry. 2015;2015:7.32.1–7.32.11. Available from: https://www.thermofisher.com/uk/en/home/life-science/cell-analysis/cell-viability-and-regulation/apoptosis/mitochondria-function/jc-1-dye-mitochondrial-membrane-potential.html. Cited 2023 Sep 1. 10.1002/0471142956.cy0732s72 25827483

[35] Varum S, Bento C, Sousa APM, Gomes-Santos CSS, Henriques P, Almeida-Santos T, et al. Characterization of human sperm populations using conventional parameters, surface ubiquitination, and apoptotic markers. Fertil Steril. 2007;87:572–83. Available from: https://linkinghub.elsevier.com/retrieve/pii/S0015028206040052. 17118365 10.1016/j.fertnstert.2006.07.1528

[36] Wang H, Sun Y, Wang L, Xu C, Yang Q, Liu B, et al. Hypoxia-induced apoptosis in the bilateral testes of rats with left-sided varicocele: a new way to think about the varicocele. J Androl. 2010;31:299–305. 20019389 10.2164/jandrol.108.007153

[37] Mohajer S, Taha RM, Ramli RB, Mohajer M. Phytochemical constituents and radical scavenging properties of Borago officinalis and Malva sylvestris. Industrial Crops Prod. 2016;94:673–81. Available from: https://linkinghub.elsevier.com/retrieve/pii/S0926669016306252.

[38] Albano Nogueira GAK, Maciel Junior VL, Minas A, Antoniassi MP. Characterization of varicocele-induced animal models: potential role of inflammasome complex in the varicocele pathophysiology. J Reprod Immunol. 2022;149:103442. Available from: https://linkinghub.elsevier.com/retrieve/pii/S0165037821001728. 34773809 10.1016/j.jri.2021.103442

[39] Razi M, Tavalaee M, Sarrafzadeh-Rezaei F, Moazamian A, Gharagozloo P, Drevet JR, et al. Varicocoele and oxidative stress: New perspectives from animal and human studies. Andrology. 2021;9:546–58. Available from: https://onlinelibrary.wiley.com/doi/10.1111/andr.12940. 33145958 10.1111/andr.12940

[40] Patki A, Shelatkar R, Singh M, Agarwal S, M V, Umbardand S, et al. Impact of antioxidants in improving semen parameters like count, motility and DNA fragmentation in sub-fertile males: a randomized, double-blind, placebo-controlled clinical trial. Translational and Clinical Pharmacology. 2023;31:28. Available from: https://tcpharm.org/x.php?id=10.12793/tcp.2023.31.e6. 10.12793/tcp.2023.31.e6 PMC10079508 37034126

[41] Li K, Yang X, Wu T. The effect of antioxidants on sperm quality parameters and pregnancy rates for idiopathic male infertility: a network meta-analysis of randomized controlled trials. Front Endocrinol. 2022;13:810242. Available from: https://www.frontiersin.org/articles/10.3389/fendo.2022.810242/full. 10.3389/fendo.2022.810242 PMC8898892 35265037

[42] Poli G, Fabi C, Sugoni C, Bellet MM, Costantini C, Luca G, et al. The role of NLRP3 inflammasome activation and oxidative stress in varicocele-mediated male hypofertility. Int J Mol Sci. 2022;23:5233. Available from: https://www.mdpi.com/1422-0067/23/9/5233. 35563625 10.3390/ijms23095233 PMC9102453

[43] Peña FJ, Johannisson A, Wallgren M, Rodriguez Martinez H. Antioxidant supplementation in vitro improves boar sperm motility and mitochondrial membrane potential after cryopreservation of different fractions of the ejaculate. Animal Reprod Sci. 2003;78:85–98. Available from: https://linkinghub.elsevier.com/retrieve/pii/S0378432003000496. 10.1016/s0378-4320(03)00049-6 12753785

[44] Samanta L, Agarwal A, Swain N, Sharma R, Gopalan B, Esteves SC, et al. Proteomic signatures of sperm mitochondria in varicocele: clinical use as biomarkers of varicocele associated infertility. J Urol. 2018;200:414–22. Available from: http://www.jurology.com/doi/10.1016/j.juro.2018.03.009. 29530785 10.1016/j.juro.2018.03.009

[45] Ammar O, Tekeya O, Hannachi I, Sallem A, Haouas Z, Mehdi M. Increased sperm DNA fragmentation in infertile men with varicocele: relationship with apoptosis, seminal oxidative stress, and spermatic parameters. Reprod Sci. 2021;28:909–19. Available from: https://link.springer.com/10.1007/s43032-020-00311-6. 32909188 10.1007/s43032-020-00311-6

[46] Jomova K, Alomar SY, Valko R, Liska J, Nepovimova E, Kuca K, et al. Flavonoids and their role in oxidative stress, inflammation, and human diseases. Chemico-Biolog Interact. 2025;413:111489. Available from: https://linkinghub.elsevier.com/retrieve/pii/S000927972500119X. 10.1016/j.cbi.2025.111489 40147618

[47] Xu YW, Ou NJ, Song YX, Wang XH, Kang JQ, Yang YJ, et al. Seminal plasma miR-210–3p induces spermatogenic cell apoptosis by activating caspase-3 in patients with varicocele. Asian J Androl. 2020;22:513. Available from: https://journals.lww.com/10.4103/aja.aja_114_19. 31670279 10.4103/aja.aja_114_19 PMC7523610

[48] Finelli R, Leisegang K, Kandil H, Agarwal A. Oxidative stress: a comprehensive review of biochemical, molecular, and genetic aspects in the pathogenesis and management of varicocele. World J Men’s Health. 2022;40:87. Available from: https://wjmh.org/x.php?id=10.5534/wjmh.210153. 34666421 10.5534/wjmh.210153 PMC8761243

[49] Chang F-W, Sun G-H, Cheng Y-Y, Chen I-C, Chien H-H, Wu G-J. Effects of varicocele upon the expression of apoptosis-related proteins. Andrologia. 2010;42:225–30. Available from: https://onlinelibrary.wiley.com/doi/10.1111/j.1439-0272.2009.00981.x. 20629644 10.1111/j.1439-0272.2009.00981.x

[50] Alam F, Syed H, Amjad S, Baig M, Khan TA, Rehman R. Interplay between oxidative stress, SIRT1, reproductive and metabolic functions. Curr Res Physiol. 2021;4:119–24. Available from: https://linkinghub.elsevier.com/retrieve/pii/S2665944121000110. 34746831 10.1016/j.crphys.2021.03.002 PMC8562188

[51] Coussens M, Maresh JG, Yanagimachi R, Maeda G, Allsopp R. Sirt1 deficiency attenuates spermatogenesis and germ cell function. Blagosklonny M, editor. PLoS ONE. 2008;3:e1571. Available from: https://dx.plos.org/10.1371/journal.pone.0001571. 18270565 10.1371/journal.pone.0001571 PMC2216432

[52] Mostafa T, Nabil N, Rashed L, Abo-Sief AF, Eissa HH. Seminal SIRT1-oxidative stress relationship in infertile oligoasthenoteratozoospermic men with varicocele after its surgical repair. Andrologia. 2020;52:e13456. Available from: https://onlinelibrary.wiley.com/doi/10.1111/and.13456. 31696601 10.1111/and.13456

[53] Agarwal A, Mulgund A, Hamada A, Chyatte MR. A unique view on male infertility around the globe. Reprod Biol Endocrinol. 2015;13:37. Available from: http://www.rbej.com/content/13/1/37. 25928197 10.1186/s12958-015-0032-1 PMC4424520

[54] Mostafa T, Rashed LA, Osman I, Marawan M. Seminal plasma oxytocin and oxidative stress levels in infertile men with varicocele. Andrologia. 2015;47:209–13. Available from: https://onlinelibrary.wiley.com/doi/10.1111/and.12248. 24635706 10.1111/and.12248

[55] Chioccarelli T, Migliaccio M, Suglia A, Manfrevola F, Porreca V, Diano N, et al. Characterization of estrogenic activity and site-specific accumulation of bisphenol-a in epididymal fat pad: interfering effects on the endocannabinoid system and temporal progression of germ cells. Int J Mol Sci. 2021;22:2540. Available from: https://www.mdpi.com/1422-0067/22/5/2540. 33802611 10.3390/ijms22052540 PMC7961766

[56] Wang Y, Chen F, Liang M, Chen S, Zhu Y, Zou Z, et al. Grape seed proanthocyanidin extract attenuates varicocele‑induced testicular oxidative injury in rats by activating the Nrf2‑antioxidant system. Molecular Medicine Reports. 2017; Available from: http://www.spandidos-publications.com/10.3892/mmr.2017.8020. 10.3892/mmr.2017.8020 29138814

[57] Palanisamy K, Krishnaswamy R, Paramasivan P, Chih-Yang H, Vishwanadha VP. Eicosapentaenoic acid prevents TCDD-induced oxidative stress and inflammatory response by modulating MAP kinases and redox-sensitive transcription factors. Br J Pharmacol. 2015;172:4726–40. Available from: https://bpspubs.onlinelibrary.wiley.com/doi/10.1111/bph.13247. 26177858 10.1111/bph.13247 PMC4594275

[58] Song Y, Wu Z, Zhao P. The protective effects of activating Sirt1/NF-κB pathway for neurological disorders. Rev Neurosci. 2022;33:427–38. 34757706 10.1515/revneuro-2021-0118

[59] Tuğcu V, Gedikbaşi A, Mutlu B, Güner E, Uhri M, Andican G, et al. Increased testicular 8-hydroxy-2’-deoxyguanosine (8-OHdG) and inducible nitric oxide synthetase (iNOS) and nuclear factor κB (NF-κB) expressions in experimental rat varicocele. Archivio italiano di urologia, andrologia : organo ufficiale [di] Societa italiana di ecografia urologica e nefrologica. 2010;82:148–53. Available from: http://www.ncbi.nlm.nih.gov/pubmed/21341550. 21341550

[60] Liu H-W, Kao H-H, Wu C-H. Exercise training upregulates SIRT1 to attenuate inflammation and metabolic dysfunction in kidney and liver of diabetic db/db mice. Nutr Metab. 2019;16:22. 10.1186/s12986-019-0349-4 PMC6446356 30988688

[61] Kisa Ü, Başar MM, Ferhat M, Çaǧlayan O. Seminal plasma Transforming Growth Factor-β (TGF-β) and Epidermal Growth Factor (EGF) levels in patients with varicocele. Turkish J Med Sci. 2008;38:105–10.

[62] Dobashi M, Fujisawa M, Yamazaki T, Okada H, Kamidono S. Distribution of intracellular and extracellular expression of transforming growth factor-beta1 (TGF-beta1) in human testis and their association with spermatogenesis. Asian journal of andrology. 2002;4:105–9. Available from: http://www.ncbi.nlm.nih.gov/pubmed/12085100. 12085100

[63] Gonzalez CR, Matzkin ME, Frungieri MB, Terradas C, Ponzio R, Puigdomenech E, et al. Expression of the TGF-beta1 system in human testicular pathologies. Reprod Biol Endocrinol. 2010;8:148. Available from: https://rbej.biomedcentral.com/articles/10.1186/1477-7827-8-148. 21126344 10.1186/1477-7827-8-148 PMC3009701

[64] Kowalewski R, Malkowski A, Sobolewski K, Gacko M. Evaluation of transforming growth factor-β signaling pathway in the wall of normal and varicose veins. Pathobiology. 2010;77:1–6. Available from: https://www.karger.com/Article/FullText/272948. 20185961 10.1159/000272948

[65] Shati AA. Resveratrol improves sperm parameter and testicular apoptosis in cisplatin-treated rats: Effects on ERK1/2, JNK, and Akt pathways. Syst Biol Reprod Med. 2019;65:236–49. Available from: https://www.tandfonline.com/doi/full/10.1080/19396368.2018.1541114. 30507263 10.1080/19396368.2018.1541114

[66] Abo El Gheit RE, Soliman NA, Nagla SA, El-Sayed RM, Badawi GA, Emam MN, et al. Melatonin epigenetic potential on testicular functions and fertility profile in varicocele rat model is mediated by silent information regulator 1. Br J Pharmacol. 2022;179:3363–81. 35064582 10.1111/bph.15804

